# Unexpected arterial thrombosis and acute limb ischemia in a young male patient with COVID-19: A case report

**DOI:** 10.3389/fsurg.2023.1092287

**Published:** 2023-01-30

**Authors:** Badr Aljabri, Mohammed Yousef Aldossary

**Affiliations:** ^1^ Division of Vascular Surgery, Department of Surgery, College of Medicine, King Saud University, Riyadh, Saudi Arabia; ^2^ Division of Vascular Surgery, Department of Surgery, Dammam Medical Complex, Dammam, Saudi Arabia

**Keywords:** acute limb ischemia, COVID-19, arterial thrombosis, thromboembolectomy, on-table angiography

## Abstract

**Introduction:**

The spread of severe acute respiratory syndrome coronavirus 2 has resulted in coronavirus disease 2019 (COVID-19) pandemic, raising significant concerns. COVID-19 can lead to thrombotic complications such as acute limb ischemia (ALI). In patients with COVID-19, thrombotic complications may increase the risk of morbidity and mortality.

**Presentation of case:**

We report the case of a 37-year-old man who presented with a 2 weeks history of right foot pain, toes blackish discoloration, and numbness. He tested positive for COVID-19 10 days prior to his presentation. Computed tomography angiography (CTA) of the lower limbs revealed near-complete occlusion of the right popliteal artery with single-vessel posterior tibial artery runoff. The patient was brought to a hybrid operating room, and diagnostic angiography confirmed the diagnosis. He underwent popliteal artery thromboembolectomy and intraoperative thrombolysis through a posterior approach. A completion angiography demonstrated a patent popliteal artery with a 2-vessels patency to the foot. His postoperative recovery was uneventful. After surgery, the popliteal, anterior tibial, and posterior tibial arteries were all palpable. The patient was discharged home on antiplatelet therapy with frequent postoperative follow-ups during the last 1 year in our outpatient clinic.

**Discussion:**

The frequency of ALI has reduced worldwide, and the hypercoagulable condition remains an infrequent cause of limb ischemia. Patients with COVID-19 have a 35%–45% thromboembolic complication rate. In many studies, the virus launches a second attack between 7 and 14 days after symptom onset, possibly causing hypercoagulability. If conservative treatment fails, various surgical methods, including thromboembolectomy, thrombolysis, and thrombosuction, can be performed to treat ALI.

**Conclusion:**

In mild ALI symptoms, unfractionated heparin can be used with vigilant follow-up. Open and endovascular procedures are currently used to treat patients with acute limb ischemia, and technological advancements continue to make interventions easier and safer.

## Introduction

Coronavirus disease 2019 (COVID-19) is a novel pandemic and has been associated with nearly 6 million deaths and 530 million confirmed infections worldwide (1). COVID-19 is more than a primary pneumonic disease as new research outlining the clinical symptoms of COVID-19 has been released. It has a systemic behavior, with thrombosis being one of the most serious complications and occurring in 31% of the patients admitted to the intensive care unit (2). Furthermore, patients with COVID-19 had a higher rate of aortoiliac thrombosis than patients without COVID-19 admitted during the same period (3). While some studies have focused on deep vein thrombosis and pulmonary embolism, evidence on arterial thrombotic events is lacking (4). With an odds ratio of 3.37 and a mortality rate of 29%, arterial thrombosis is an emerging diagnosis in the COVID-19 pandemic (5, 6). In this study, we present a case of right acute limb ischemia (ALI) due to acute arterial thrombosis in a 37-year-old man diagnosed with COVID-19 and treated with open thromboembolectomy.

## Case description

A 37-year-old man with non-significant past medical history presented to the emergency department with right foot pain, toes progressive bluish discoloration, and numbness for 2 weeks. The pain described as sudden, continuous, and associated with coldness and numbness in the same limb. He denied any history of smoking or alcohol abuse. Past medical history and surgical history were unremarkable.

Ten days prior to this presentation, he was tested positive for COVID-19 after minor upper respiratory tract infectious symptoms and was home quarantined. He had no personal or family history of hematological disorders.

**Figure 1:**
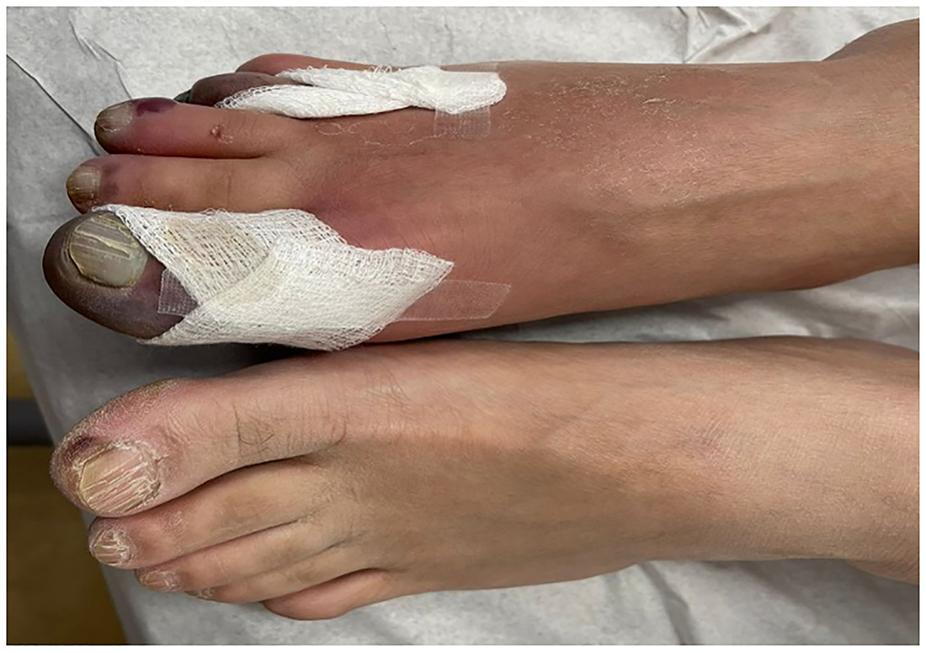
Local examination of the right foot revealed mild edema and erythema and blackish discoloration of the first and fourth toes.

His clinical presentation was believed to be a thrombotic complication of COVID-19. Upon general physical examination, the patient was in severe pain, but vital signs were stable. Local examination of the right foot revealed mild edema and erythema, blackish discoloration of the first and fourth toes, and severe tenderness on palpation (Figure 1). Pulse examination revealed palpable femoral and popliteal pulses and the absence of dorsalis pedis and posterior tibial pulses with detectable monophasic Doppler signals. Examination of the other limb was unremarkable.

## Diagnostic assessment

Laboratory examination revealed mild leukocytosis and thrombocytosis (platelet count, 522 × 109/L), and D-Dimer, 0.43 μ/ml. The antineutrophil cytoplasmic antibody, antinuclear antibody, rheumatoid factor, cryoglobulin, hepatitis panel, cytomegalovirus test, thrombophilia screening, human immunodeficiency virus test, and syphilis serology were all negative. The levels of homocysteine, C-reactive protein, liver and renal function, erythrocyte sedimentation rate, and coagulation profile were all within the normal range.

**Figure 2:**
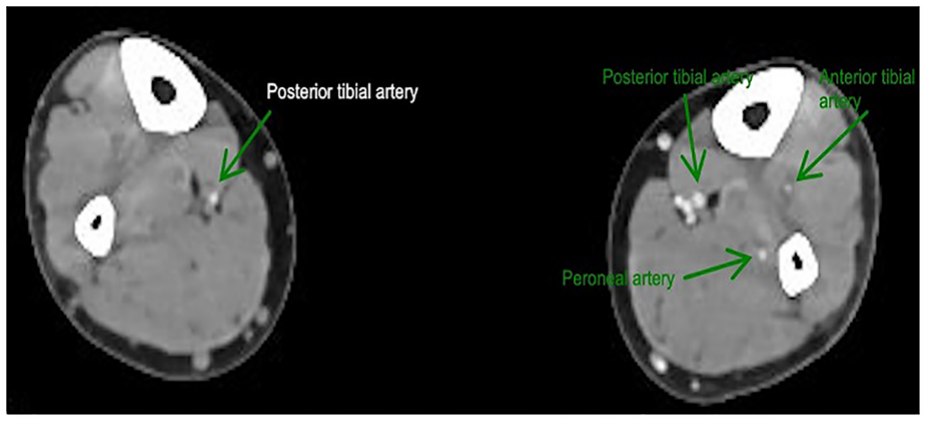
CTA of the lower limbs axial view showed right single-vessel posterior tibial artery runoff.

Computed tomography angiography (CTA) of the lower limbs revealed near-complete occlusion of the right popliteal artery behind the knee joint with single-vessel posterior tibial artery runoff (Figure 2).

**Figure 3:**
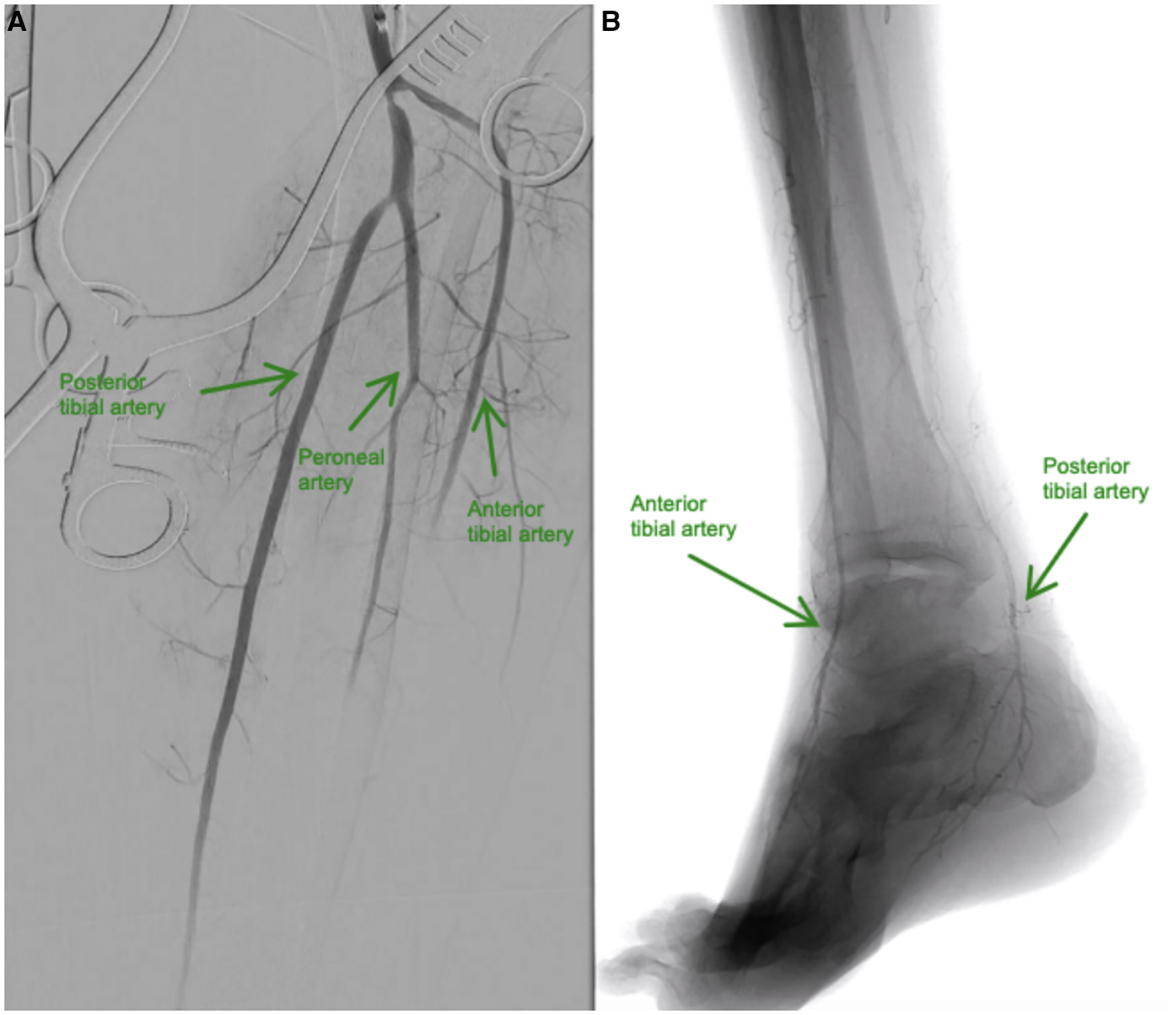
(**A** and **B**). (**A**) Baseline angiography shows total occlusion from the mid-anterior tibial artery to the dorsalis pedis artery, and total occlusion of the distal posterior tibial and mid-peroneal arteries. (**B**) Resolution of blood flow after intra-arterial infusion of alteplase in the anterior tibial and posterior tibial arteries.

Immediately, 5000 IU of unfractionated heparin (UFH) bolus was intravenously administered followed by continuous UFH infusion; however, foot pain persisted despite adequate analgesics. Consequently, the patient was taken to the hybrid operating room for diagnostic angiography with surgical thrombectomy and intraoperative thrombolysis. Through a posterior approach, the popliteal artery and its bifurcation were dissected and controlled; an arteriotomy was performed at the popliteal artery. Thromboembolectomy was performed using a Fogarty catheter, and then direct intra-arterial injection of alteplase 40 mg slowly over 60 min *via* a multi-side hole catheter into the anterior tibial and tibioperoneal trunk was performed. A completion angiography revealed a patent popliteal artery with a 2-vessels patency to the foot (Figure 3A,B).

**Figure 4:**
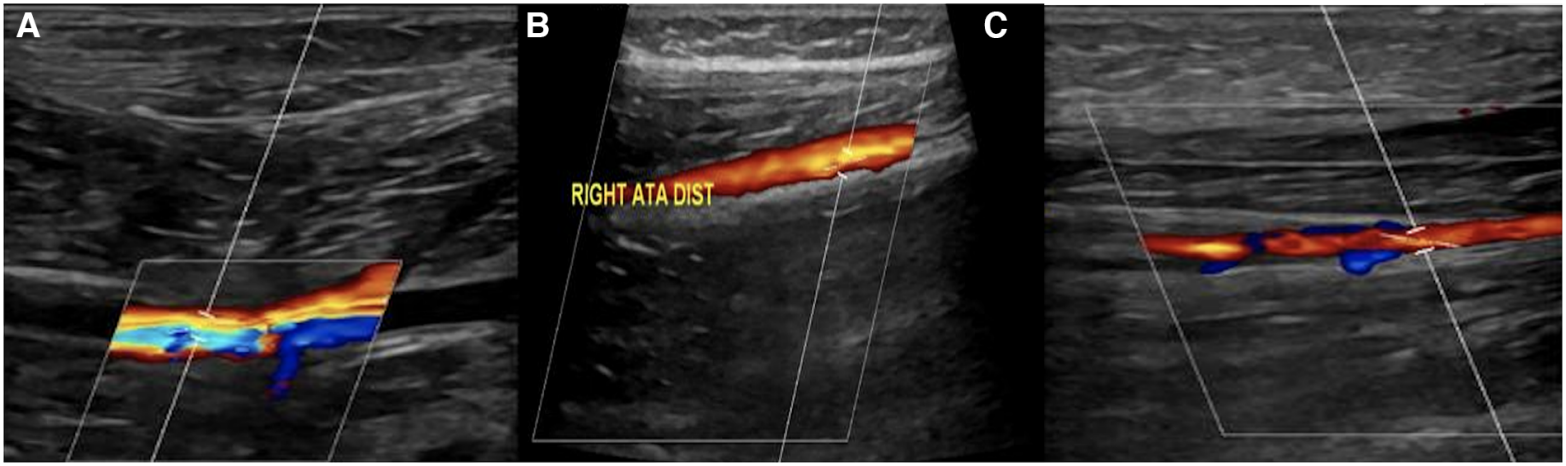
(**A, B**, and **C**): right lower limb arterial duplex ultrasound showed patent (**A**) right popliteal artery, (**B**) distal anterior tibial artery, (**C**) and posterior tibial artery.

The popliteal artery was repaired using a bovine pericardial patch. The postoperative recovery was uneventful. After surgery, the popliteal, anterior tibial, and posterior tibial arteries were all palpable. The UFH infusion continued for 7 days. The neurological symptoms and pain of ALI completely resolved 3 weeks after the procedure. The patient was discharged home on antiplatelet therapy. A follow-up lower limb arterial duplex ultrasound after 3 months showed patent right popliteal, distal anterior tibial, and posterior tibial arteries (Figure 4A–C).

## Discussion

Although the overall frequency of ALI has declined worldwide, the hypercoagulable condition remains an infrequent cause of limb ischemia (7). Patients with COVID-19 have a thromboembolic complication rate of 35%–45% (8). A high incidence of both venous and arterial thromboembolisms was reported in patients with critical illness, which is linked to a higher mortality rate (2, 9).

Patients with COVID-19 have a higher risk of thrombotic events such as strokes than patients in general wards (10). However, several reasons may have contributed to this relative rise in arterial thrombotic events during the pandemic, including delays in emergency room presentation because of lockdown, older patient age, or fear of going to hospitals due to a high risk of contamination (11). Furthermore, some studies have reported that these thrombotic events occur at a later stage in the infection (12). Some authors believe that the virus launches a second attack between 7 and 14 days after symptom onset, possibly causing hypercoagulability (13). In this patient, he had arterial thrombosis 10 days after diagnosis with COVID-19.

Similarly, other investigators have reported that patients with thrombotic complications are typically young and have no history of significant atherosclerosis seen by computed tomography or angiography (14). Thus, this finding implies that a significant proportion of arterial thromboses in patients with COVID-19 occur in unaffected arteries or arteries with mild damage.

ALI appears to be caused by a systemic inflammatory process driven by numerous activated macrophages that generate a cytokine storm (15). COVID-19 induces an increase in cytokine levels, including tumor necrosis factor, interleukin-1, interleukin-6, procalcitonin, and interferon (16–18). Patients with COVID-19 are more likely to have thrombotic dysfunction, and those with severe symptoms had increased C-reactive protein levels and higher thrombotic risk (19).

Evidence shows that severe acute respiratory syndrome coronavirus 2 induces a procoagulant condition that results in both micro and macrothrombi (20). Even in the absence of major artery thrombosis, cutaneous ischemia lesions are common in these patients (21). Patients with COVID-19 have shown considerable endothelial injury, extensive thrombosis with microangiopathy, alveolar–capillary microthrombi, and new growing vessels (22). Partial vascular endothelial shedding, vascular intimal inflammation, and thrombosis are some of the vascular pathological changes seen in these patients (23).

ALI is a limb-threatening thromboembolic event that requires emergent surgery. If surgery is required, various techniques are available, including thromboembolectomy, thrombolysis, and thrombosuction, with comparable results in terms of limb salvage (24). The decision to perform surgery is influenced by the clinical condition of the patient and ALI etiology.

The operative strategy should include a selective, on-table angiography. On-table angiography is used if intraoperative situation suggested an unanticipated problem. With rare exceptions, recent guidelines recommend routine completion angiography following the procedures (25). Despite the weak evidence quality, this recommendation (Class 1) is strong (Grade C).

Patients with COVID-19 may develop ALI, thrombotic events such as venous thromboembolism, thrombotic stroke, and acute myocardial infection. In mild ALI symptoms, unfractionated heparin can be used with vigilant follow-up. Open and endovascular procedures are currently used to treat patients with acute limb ischemia, and technological advancements continue to make interventions easier and safer.

## Data Availability

The raw data supporting the conclusions of this article will be made available by the authors, without undue reservation.
